# Effects of *Schisandra chinensis* Polysaccharide-Conjugated Selenium Nanoparticles on Intestinal Injury in Mice

**DOI:** 10.3390/ani13050930

**Published:** 2023-03-04

**Authors:** Hongxu Du, Xiaoyan Tan, Zhangxun Li, Hong Dong, Lijuan Su, Zhengke He, Qi Ma, Shiqi Dong, Mythili Ramachandran, Juan Liu, Liting Cao

**Affiliations:** 1 Department of Traditional Chinese Veterinary Medicine, College of Veterinary Medicine, Southwest University, Chongqing 402460, China; 2 Beijing Key Laboratory of Traditional Chinese Veterinary Medicine, Beijing University of Agriculture, Beijing 102206, China; 3 National Center of Technology Innovation for Pigs (NCTIP-XD/C17), Chongqing 402460, China; 4 Immunology Research Center, Medical Research Institute, Southwest University, Chongqing 402460, China; 5 Department of Biochemistry and Molecular Medicine, University of California Davis, Sacramento, CA 95817, USA

**Keywords:** *Schisandra chinensis* polysaccharide, selenium nanoparticle, intestinal inflammatory injury

## Abstract

**Simple Summary:**

Animal enteritis caused by Gram-negative bacterial infection is one of the most common and harmful diseases in the livestock and poultry industry. *Schisandra chinensis* polysaccharide (SCP) has potential effects on the treatment of intestinal injury. Selenium nanoparticle modification can improve the bioactivity of polysaccharides. In this study, SCP-conjugated selenium nanoparticles (SCP-Se NPs) were firstly synthesized, and their effect on intestinal injury was also investigated. The results showed that SCP-Se NPs could more effectively alleviate LPS-induced diarrhea, intestinal tissue injury, and tight junction destruction and decrease the elevated inflammatory cytokine expression levels of TNF-α, IL-1β, and IL-6 compared with SCP in mouse models, suggesting that SCP-Se NPs can serve as a good candidate for preventing and treating enteritis in the livestock and poultry industry.

**Abstract:**

*Schisandra chinensis* polysaccharide (SCP) is an experimental therapeutic for the treatment of intestinal injury. Selenium nanoparticle modification can improve the bioactivity of polysaccharides. In this study, SCP was firstly extracted and purified by a DEAE-52 column, then SCP-Selenium nanoparticles (SCP-Se NPs) were prepared, and the procedure was optimized. Thereafter, the obtained SCP-Se NPs were characterized by transmission electron microscope, X-ray diffraction, energy-dispersive X-ray spectroscopy, and Fourier transform infrared spectroscopy. The influence of different storage environments on the stability of colloidal SCP-Se NPs was also investigated. Finally, the therapeutic effects of SCP-Se NPs on LPS-induced intestinal inflammatory injuries in mice were evaluated. Results showed that the optimized SCP-Se NPs were amorphous, uniform, spherical particles with a diameter of 121 nm, and the colloidal solution was stable at 4 °C for at least 14 d. Moreover, SCP-Se NPs could more effectively alleviate LPS-induced diarrhea, intestinal tissue injury, and tight junction destruction and decrease the elevated expression levels of TNF-α, IL-1β, and IL-6 compared with SCP. These results demonstrate that SCP-Se NPs may alleviate LPS-induced enteritis through their anti-inflammatory effects, indicating that SCP-Se NPs can serve as a good candidate for preventing and treating enteritis in the livestock and poultry industry.

## 1. Introduction

The intestinal tract is the main organ for the digestion and absorption of nutrients [1,2]. Therefore, its health status directly determines the nutritional supply and overall growth of the animal. The intestinal tract is also a crucial barrier for defending against external pathogenic factors. Animal enteritis, caused by Gram-negative bacterial infection, is one of the most common and harmful diseases in the livestock and poultry industry [3,4]. In production practice, antibiotic treatment is the current standard of care for enteritis. However, with the rise of drug residues, antibiotic resistance, and food safety concerns, the development of new safe antibacterial alternatives to alleviate animal enteritis has become an urgent matter for the development of the current livestock and poultry industry.

Traditional Chinese veterinary medicine has a long history of safeguarding the health of livestock and poultry in China and other Asian countries [5,6]. *Schisandra chinensis* polysaccharide (SCP) is one of the main active components of the traditional Chinese medicine *Schisandra chinensis* (Turcz.) Baill and has beneficial therapeutic potential for a variety of enteritis diseases [7,8]. For example, SCP has been reported to alleviate antibiotic-associated enteritis caused by lincomycin hydrochloride by inhibiting pro-inflammatory factors and increasing the expression of anti-inflammatory factors [9]. Additionally, SCP also has a therapeutic effect on mice with ulcerative colitis by restoring the intestinal structure, regulating cytokine levels, and improving the diversity of intestinal flora [10]. However, due to the macromolecular structure of the polysaccharide, its bioavailability is extremely low, which limits its clinical application.

Selenium is an essential trace element for animals, as shown with its anti-inflammatory, antioxidant, and antiviral properties [11,12,13,14]. Selenium supplementation can alleviate intestinal inflammation, improve the structure of intestinal flora, and enrich the diversity of intestinal flora [15,16,17,18]. Currently, inorganic and organic selenium are important sources of selenium supplements, but the narrow therapeutic window limits its clinical application [19].

As a new form of selenium, nano-selenium has attracted much attention for its higher biological activity and safety compared with traditional inorganic and organic selenium [20,21]. However, due to high surface energy, bare selenium nanoparticles (Se NPs) quickly aggregate into black or gray elemental selenium, reducing their bioactivity, biocompatibility, and bioavailability [22]. It has been reported that polysaccharide molecules can react with the surface of Se NPs to form stable amorphous nanoparticles through intermolecular hydrogen bonds, seleno-oxygen bonds, and selenium-nitrogen bonds [23]. The polysaccharide-Se NPs conjugate preparation using the polysaccharide as a soft template solves the drawback of easy agglomerative inactivation of Se NPs. It also improves the bioavailability and bioactivity of polysaccharides by nanosizing and selenization [24]. Hence, the research on polysaccharide-based Se NPs derivatives has become a hot spot in the field of polysaccharide and nanomedicine in recent years. For example, it was found that the nano-selenium derivative of Ulva polysaccharide significantly enhanced its therapeutic effect on DSS-induced ulcerative colitis in mice by inhibiting the pro-inflammatory response mediated by the NF-κB pathway [25]. However, whether SCP-Se NPs can be synthesized by using SCP as a template and whether the synthesized SCP-Se NPs have an effect on the intestinal injury induced by LPS in mice has not been reported.

In this study, considering the bioactivities of SCP, we firstly extracted and purified SCP and then optimized the synthesis procedure of SCP-Se NPs using the particle diameter, polydispersity index (PDI), and Zeta potential examination analyzed by ultraviolet-visible (UV-Vis) spectroscopy and dynamic light scattering (DLS). Transmission electron microscopy (TEM), X-ray diffraction (XRD), energy-dispersive X-Ray spectroscopy (EDX), and Fourier transform infrared (FTIR) spectroscopy were used to further characterize the prepared SCP-Se NPs colloidal solution. Additionally, the stability of the colloidal solution in different storage conditions was studied. Finally, the intestinal protective effects of SCP-Se NPs were investigated based on the disease activity index (DAI) scoring analysis, hematoxylin–eosin (HE) staining, immunohistochemical (IHC) staining, and qRT-PCR examination of the pro-inflammation index in vivo.

## 2. Materials and Methods

### 2.1. Reagents and Materials

*Schisandra chinensis* (Turcz.) Baill was obtained from Chongqing Zhong Miao medicine Co., Ltd. Lipopolysaccharide (LPS, *Escherichia coli* 055:B5), DAB substrate kit was purchased from Biosharp Beijing Labgic Technology Co., Ltd., Beijing, China. Hematoxylin–eosin staining solution was purchased from Changde BKMAM Biotechnology Co., Ltd., Changde, China. Bovine serum albumin (BSA) and DEAE-52 cellulose were purchased from Beijing Solarbio Science & Technology Co., Ltd., Beijing, China. Monosaccharide references (mannose, ribose, rhamnose, glucuronic acid, galacturonic acid, glucose, xylose, and arabinose) were purchased from Shanghai baomanbio Co., Ltd., Shanghai, China. Anti-ZO-1 rabbit polyclonal antibody and HRP-labeled goat anti-rabbit IgG antibody were purchased from Wuhan Servicebio Biotechnology Co., Ltd., Hubei, China. IL-1β, IL-6, and TNF-α Elisa kits were obtained from Multi Sciences Biotech, Co., Ltd., Hangzhou, China. RNAiso Plus, PrimerScript^TM^ RT reagent kit with gDNA Eraser, and SYBR^®^ Premix Ex Taq^TM^ II (Tli RNaseH Plus) kit were purchased from Takara Biomedical Technology (Beijing) Co., Ltd., Beijing, China. Sodium selenite, ascorbic acid, ethyl alcohol, xylene, paraformaldehyde, trifluoroacetic acid, 1-phenyl-3-methyl-5-pyrazolone, chloroform, and other reagents were purchased from Chengdu Cologne Chemical Co., Ltd., Chengdu, China.

### 2.2. Isolation and Purification of SCP

SCP was extracted from *Schisandra Chinensis* (Turcz.) Baill according to methods described by previous studies [26]. Briefly, dried *Schisandra chinensis* (Turcz.) Baill was defatted with 95% ethyl alcohol for 24 h, dehydrated with a drying oven, and boiled with distilled water (1:20, w/v) at 100 °C three times (2 h each time). After the supernatant was filtrated, collected, and concentrated with rotary evaporation at 60 °C under reduced pressure, three volumes of ethyl alcohol were slowly added and kept overnight at 4 °C. Subsequently, the precipitate was collected, dissolved with distilled water, and subjected to protein removal by the Sevage method. Then, the polysaccharide was further purified by using a DEAE-52 cellulose column (2.50 × 30.0 cm) and eluted with a 0.200 M distilled water and 0.400 M NaCl solution at a flow rate of 1.00 mL/min. Each 10.0 mL fraction was collected and monitored by the phenol-sulfuric acid method [27] to plot the elution curve. The eluents in the same fraction were collected, combined, and dialyzed against distilled water within a molecular weight cutoff 3500 Da dialysis bag for 48 h. Finally, the purified SCP was concentrated and lyophilized with a lyophilizer (Scientz-10N, Ningbo Xinzhi Freeze-drying Equipment Co., Ltd., Ningbo, China).

### 2.3. Monosaccharide Composition Analysis of SCP

The monosaccharide composition analysis of SCP was performed according to previous studies [28]. Briefly, SCP was hydrolyzed with trifluoroacetic acid (TFA, 4 M), and the hydrolysate was added to 1-phenyl-3-methyl-5-pyrazolinone (PMP) solution (0.500 M, dissolved in methanol) for derivation and filtered by 0.450 μm filter for later use. Analysis was performed using a liquid chromatography pump (LC-20AD Shimadzu, Japan) under the following conditions: Shim-pack vp-ods C18 column (250 mm × 4.60 mm, 5.00 μm), column temperature 30 ℃, mobile phase—acetonitrile: phosphate buffer = (17:83), flow rate of 1.00 mL/min, UV detector (spd-20a shimadzu, Kyoto, Japan), detection wavelength of 245 nm, injection volume of 10.0 μL, and acquisition time of 60 min.

### 2.4. Preparation of SCP-Se NPs

**Figure 1:**
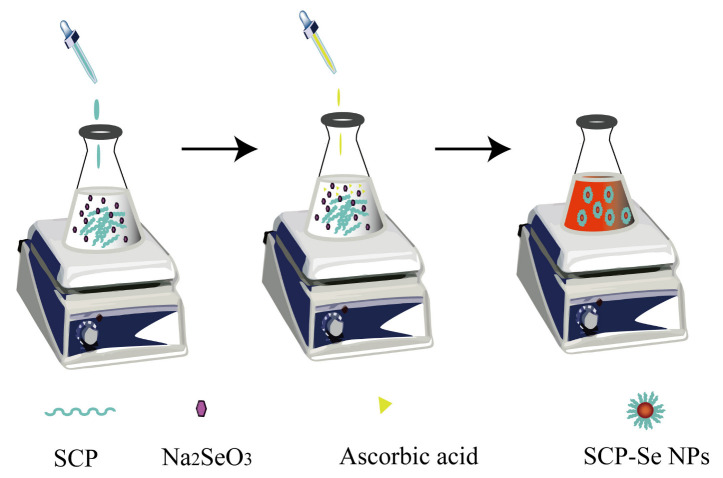
The schematic diagram of SCP-Se NPs preparation.

SCP-Se NPs were prepared by the Na_2_SeO_3_/ascorbic acid chemical reduction approach. Based on the reaction principle and referring to previous relevant studies [22,29], the molar ratio of Na_2_SeO_3_ to ascorbic acid was fixed at 1:4. Firstly, a 3.00 mL SCP solution of different concentrations was mixed with 0.500 mL Na_2_SeO_3_ solution (50.0 mM) for 10 min, respectively. Subsequently, 1.00 mL of ascorbic acid solution (100 mM) was slowly added to the above mixture under magnetic stirring at a speed of 1200 rpm. After reacting for 3 h at room temperature (RT) in the dark, the final reaction solution was dialyzed with a dialysis bag against ultra-pure water until no Se was detected in the outer solution. Finally, the SCP-Se NPs colloidal solution was obtained and kept at 4 °C for the following experiments. The schematic diagram is shown in Figure 1.

### 2.5. Optimization of SCP-Se NPs Synthesis

The obtained SCP-Se NPs were characterized by using multiple methods. Since SCP-Se NPs solution has the basic colloidal characteristics, dual-wavelength spectrophotometry was first carried out to monitor the particle size according to previous research [30,31]. In brief, the absorbance of the SCP-Se NPs solution was measured at 410 nm and 490 nm by using a G-9 Series double beam UV-Vis spectrophotometer (G-9S, Nanjing FILA Instrumen, Nanjing, China), and the *A*_410_/*A*_490_ value was calculated. Meanwhile, the index of particle diameter, PDI, and Zeta potential were also determined by a DLS analyzer (Litesizer 500, Anton Paar, Graz, Austria).

### 2.6. Characterization of SCP-Se NPs

To observe the morphology and distribution of SCP-Se NPs directly, TEM (JEM-2800, JEOL, Akishima, Japan) was applied. Briefly, 20.0 μL of the SCP-Se NPs colloidal solution was placed on the porous carbon film surface of the copper mesh and dried in an oven to avoid contamination by dust and other impurities. After drying, the sample area was observed by TEM.

The crystal form of SCP-Se NPs was measured with an X-ray diffractometer from 5° to 90° at an angle of 2^θ^ and a speed of 10°/min with the operating current and voltage set at 30 mA and 40 kV, respectively.

The SCP-Se NPs sample was put into the test window, and the energy-dispersive X-ray spectroscopy was started to obtain the SCP-Se NP element analysis results.

The infrared absorption spectra of SCP and SCP-Se NPs were determined by an infrared spectrometer (iS50 FTIR, Thermo Nicolet Corporation, Waltham, MA, USA). Briefly, dried samples were mixed with potassium bromide solid powder (spectrally pure grade) in a ratio of 1/100, thoroughly ground in a mortar, transferred to a mold, and made into sheets for analysis in the spectral range of 4000–400 cm^−1^.

### 2.7. Stability of SCP-Se NPs Colloidal Solution at Different Storage Conditions

The stability of the SCP-Se NPs colloidal solution at different storage temperatures was determined by visual changes and the detection of particle diameter size, PDI, and Zeta potential. The SCP-Se NPs colloidal solution samples stored at 4 °C and RT were observed, and the particle size, PDI, and Zeta potentials of the SCP-Se NP samples were measured on days 0, 3, 7, and 14.

### 2.8. Anti-Intestinal Inflammatory Injury Activity of SCP-Se NPs In Vivo

#### 2.8.1. Animal Feeding and Management

Twenty-eight SPF male KM mice were obtained from Southwest Medical University Laboratory Animal Center. During the whole experiment, all the mice were housed in the Animal Experimental Center of Southwest University at a temperature of 25 °C, relative humidity of 50%, and a light and dark cycle of 12 h, and they had adaptive feeding for one week before the experiment. Meanwhile, the mental state, activity, diet, urine, and feces of the mice were observed every day. The animal study was conducted in accordance with the guidelines approved by the Southwest University Laboratory Animal Welfare Ethics Committee (Ethics NO.: IACUC-20210915-03).

#### 2.8.2. Effects of SCP-Se NPs on LPS-Induced Enteritis

After adaption, the mice were randomly divided into four groups: blank control group (BC group), LPS model group, SCP group, and SCP-Se NPs group. After 14 days of intragastric administration of the corresponding solutions (SCP group—10.0 mg/kg SCP solution; SCP-Se NPs group—10.0 mg/kg SCP-Se NPs solution [32]; and BC group and LPS group—equal volume of sterile water), mice in the LPS, SCP, and SCP-Se NPs groups were intraperitoneally injected with 20.0 mg/kg LPS solution [33], and the BC group was intraperitoneally injected with the equal volume of sterile saline. Thereafter, body weight, fecal occult blood, and diarrhea of mice within 6 h after intraperitoneal administration were observed and recorded for DAI score analysis [34]. At the end of the experiment (6 h post-LPS injection), the mice in the different groups were sacrificed under anesthesia, serum was collected, part of the jejunum tissue was fixed in 4% paraformaldehyde solution, and the other part was rapidly collected and frozen in liquid nitrogen.

#### 2.8.3. Histopathological Analysis of Jejunum

After being fixed in 4% paraformaldehyde solution for 24 h, the jejunum tissue samples were dehydrated with gradient alcohol solutions, made transparent with xylene, embedded in paraffin, and sliced into sections of 4.00 μm thickness by a microtome (LEICA RM2016, Wetzlar, Germany). Subsequently, the slices were stained with HE-staining solution, and images were taken under an optical microscope (ZEISS Axiocam ERc 5S, Oberkochen, Germany). Meanwhile, the villi height (from the apex of the villi to the junction of the villi crypt) and crypt depth (the depth of the invagination between adjacent villi) were measured with the ZEISS image analysis system. Values are means from 20 complete villi, and only vertically oriented villi and crypts from each slide were measured.

#### 2.8.4. IHC Staining

Paraffin-embedded tissues were prepared as described in Section 2.8.3, cut into 4.00 μm thick sections, dewaxed with xylene, rehydrated with gradient alcohol solutions, and sealed with 5% BSA. Then, sections were incubated with the anti-ZO-1 antibody at 4 °C overnight and with HRP-labeled goat anti-rabbit IgG at RT for 30 min. Finally, the DAB working solution was used for color rendering, and the hematoxylin solution was incubated for 5 min. The images were analyzed after capture using ImageJ and the IHC Profiler plugin, and six different images were counted for each group. The average gray value (staining intensity) and percentage of the positive area (staining area) of positive cells were taken as IHC measurement indicators, and the final scores were given.

#### 2.8.5. Inflammatory Cytokine Analysis

To investigate the effect of different treatments to alleviate intestinal inflammatory injury, the serum cytokine contents and the relative mRNA expression levels of jejunum IL-1β, IL-6, and TNF-α were detected. The serum cytokine contents were tested by ELISA kits according to the manufacturer’s instructions. The relative mRNA expression levels of the inflammatory cytokines were detected using qRT-PCR method. Briefly, total RNA was extracted from jejunum tissue homogenate with an RNAiso Plus kit. After the concentration was determined by BioDrop μLITE (BioDrop, UK), cDNA was synthesized using a PrimerScript^TM^ RT Reagent kit with gDNA Eraser. Hereafter, the mRNA expression level of each gene was determined by using a QuantStudio 3 real-time fluorescence quantitative PCR instrument (Thermo Fisher Scientific Co., Ltd. New York), and the relative gene expression level of each target gene was calculated by the 2^-ΔΔCt^ method. The primer sequences used in this experiment are shown in Table 1. The reaction procedures are as follows: 95 °C for 30 s, followed by 40 cycles at 95 °C for 5 s, and 60 °C for 30 s. All these above operations were strictly in accordance with the instructions.

### 2.9. Statistical Analysis

All results were expressed as “Mean ± SD” from at least three independent experiments. The differences between multiple groups were analyzed by Duncan’s multiple range test using SPSS 20.0 analysis software. Differences were considered significant at *p* < 0.05.

## 3. Results

### 3.1. Isolation and Purification of SCP

**Figure 2:**
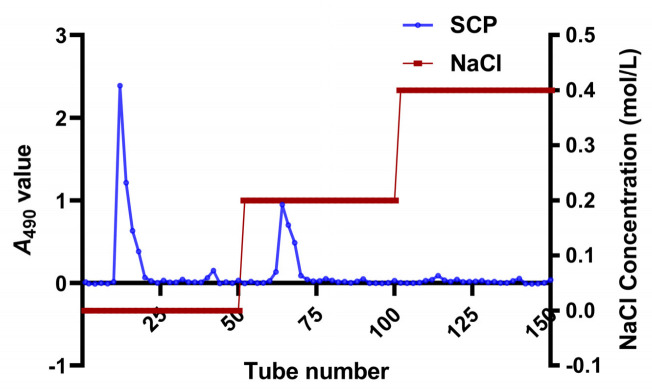
The elution curve of SCP on DEAE-52 cellulose column.

In this study, SCP was successfully extracted by the water extraction and alcohol precipitation method and purified with DEAE-52 cellulose column chromatography. As shown in Figure 2, two main fractions were obtained, of which the elution curves were single peaks in distilled water and 0.200 M NaCl eluents. Meanwhile, there was no obvious elution peak in the 0.400 M NaCl eluent. Therefore, eluents of tubes 12–18 and 62–68 were collected and combined. After dialysis and lyophilization, the purity of SCP was determined using the carbohydrate content of 89.2%.

### 3.2. Monosaccharide Composition of SCP

**Figure 3:**
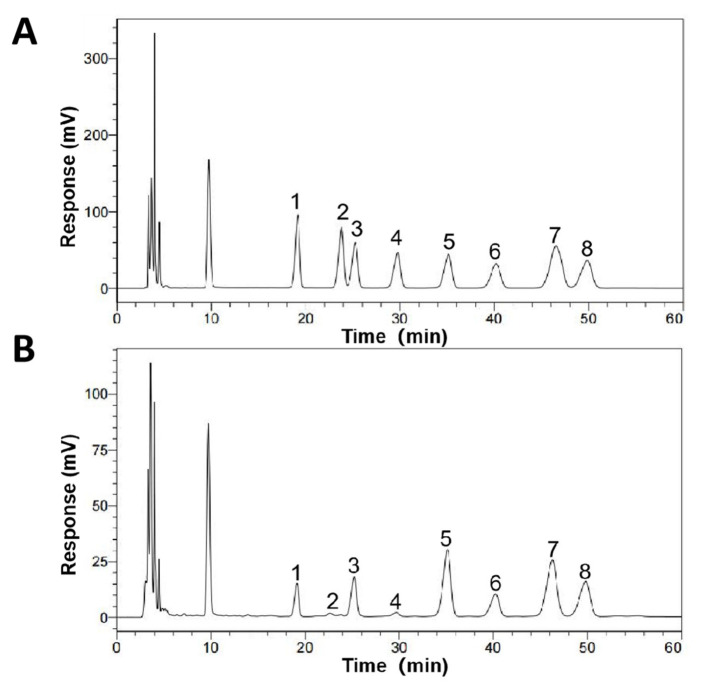
Monosaccharide composition analysis of SCP by HPLC. (**A**) HPLC chromatograms of mixed standards. (**B**) HPLC chromatograms of SCP. Monosaccharide corresponding to each peak: 1. mannose, 2. ribose, 3. rhamnose, 4. glucuronic acid, 5. galacturonic acid, 6. glucose, 7. xylose, 8. arabinose.

The monosaccharide composition results of SCP are shown in Figure 3. SCP was composed of mannose, ribose, rhamnose, glucuronic acid, galacturonic acid, glucose, xylose, and arabinose with the mole ratio of 6.93:1.00:14.7:1.71:29:13.0:21.3:23.1.

### 3.3. Preparation and Characterization of SCP-Se NPs

**Figure 4:**
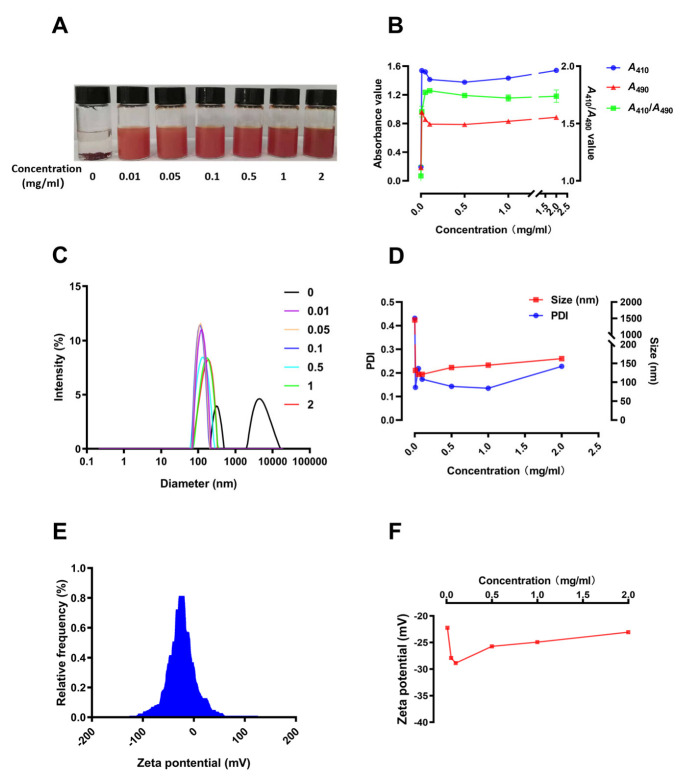
Optimization of SCP-Se NPs preparation process. (**A**) The appearance of SCP-Se NPs modified with different concentrations of SCP. (**B**) The A410, A490, and A410/A490 values of SCP-Se NPs solution at different SCP concentrations. (**C**) The particle size distribution of SCP-Se NPs at different SCP concentrations. (**D**) The median particle size and PDI of SCP-Se NPs at different SCP concentrations. (**E**) Zeta potential of SCP solution. (**F**) Zeta potential of SCP-Se NPs at different SCP concentrations.

To investigate the appropriate SCP concentration for SCP-Se NPs formation, 0 mg/mL, 0.0100 mg/mL, 0.0500 mg/mL, 0.100 mg/mL, 0.500 mg/mL, 1.00 mg/mL, and 2.00 mg/mL of SCP solution were added to the reaction system, respectively. As shown in Figure 4A, Se NPs quickly aggregated and precipitated when there was no SCP in the system and when the Se NPs solution with SCP modification was orange-red and evenly dispersed. Meanwhile, the absorbance values of the solution prepared with different SCP concentrations were also measured, and the values of *A*_410_/*A*_490_ were calculated (Figure 4B). When SCP concentration reached 0.100 mg/mL, the value of *A*_410_/*A*_490_ was the highest and tended to be more stable at higher concentrations. Figure 4C,D shows the particle size distribution, PDI, and median particle size of the Se NPs prepared by different SCP concentrations. The particle size distribution of the bare Se NPs was polydisperse with a PDI of 0.430 and a median particle size of 1453 nm. However, the uniformity of the solution greatly changed when SCP was added to the reaction system. Especially for 0.100 mg/mL SCP, the nanoparticles showed the best uniformity with the PDI of 0.170 and the median particle size of 121 nm. Figure 4E,F illustrates the Zeta potential of SCP and SCP-Se NPs modified with different concentrations of SCP. As shown in Figure 4E, the Zeta potential of SCP was −28.9 mV. With the increase of the SCP concentration, the Zeta potential of the SCP-Se NPs first increased and then decreased (Figure 4F). Moreover, at the SCP concentration of 0.1 mg/mL, SCP-Se NPs showed the highest potential of −28.9 mV.

### 3.4. Characterization of SCP-Se NPs

**Figure 5:**
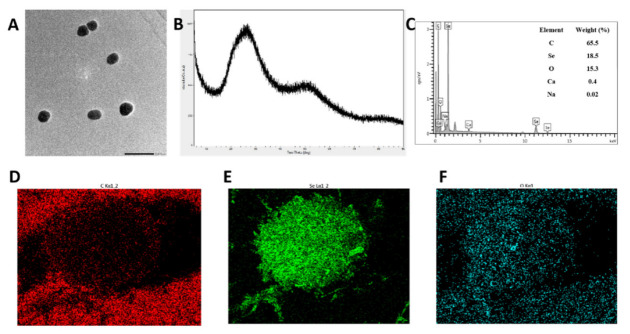
SCP-Se NP characterization. (**A**) TEM image of SCP-Se NPs. (**B**) XRD spectrum of SCP-Se NPs. (**C**) The elemental composition of SCP-Se NPs. (**D**) Carbon. (**E**) Selenium. (**F**) Oxygen.

Based on the above experiments, 0.100 mg/mL SCP was identified as the optimal concentration for SCP-Se NP preparation. TEM images showed that SCP-Se NPs presented a monodisperse uniform spherical structure with a particle diameter of almost 120 nm (Figure 5A), similar to the above results shown in Figure 4D. The XRD result showed that there was only a broad diffraction peak at lower angles (Figure 5B), indicating that SCP-Se NPs were amorphous according to the JCPDS database. Furthermore, the elemental composition of SCP-Se NPs was also analyzed by EDX, as shown in Figure 5C–F. It was found that five signals were obtained, in which the proportion of carbon was 65.5%, followed by selenium (18.5%), oxygen (15.3%), calcium (0.400%), and sodium (0.0200%).

**Figure 6:**
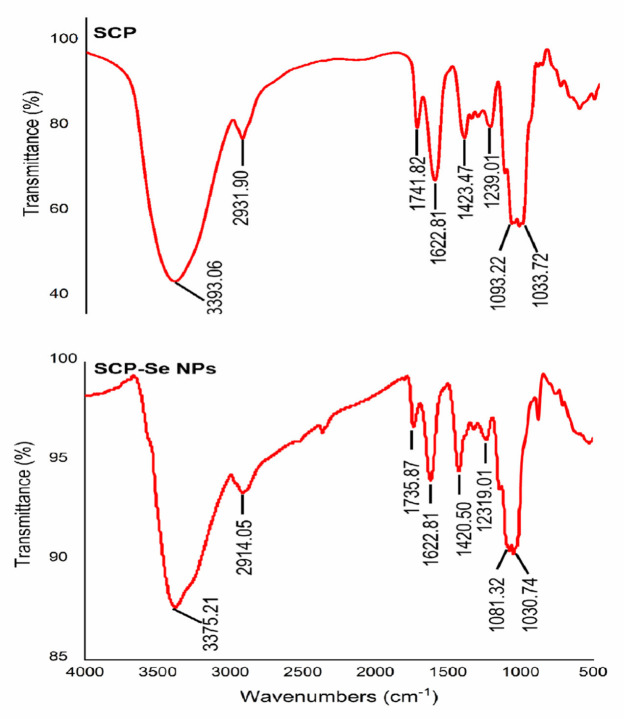
FTIR spectra of SCP and SCP-Se NPs.

To clarify the possible banding mechanism between selenium and SCP, FTIR was employed to provide information on their interaction. As shown in Figure 6, SCP and SCP-Se NPs had similar spectra. The absorption peaks of SCP at 3393 cm^−1^, 2932 m^−1^, and 1623 cm^−1^ represent the stretching vibration of the O-H and C-H groups, which are the characteristic peaks of polysaccharides. It could be clearly seen that the characteristic absorption peaks of the O-H and C-H groups for SCP shifted to lower wavelengths of 3375 cm^−1^ and 2914 cm^−1^ for SCP-Se NPs. Meanwhile, the absorption peak at 1093 cm^−1^ of SCP belonging to the C-O group stretching vibration was slightly shifted to 1081 cm^−1^ in the SCP-Se NPs spectrum. These results may indicate that the interaction between O-H and C-O of SCP with the surface of Se NPs forms new O-H···Se bonds and C-O···Se bonds.

### 3.5. Stability of SCP-Se NPs at Different Storage Temperatures

**Figure 7:**
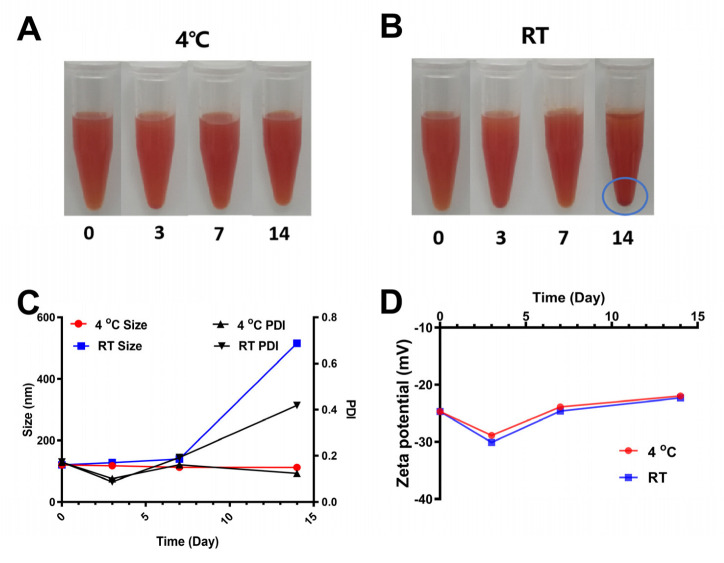
Stability of SCP-Se NPs at different storage temperatures. (**A**) Appearance changes of SCP-Se NPs colloidal solution at 4 °C. (**B**) Appearance changes of SCP-Se NPs colloidal solution at room temperature (RT). (**C**) Changes in particle size and PDI of SCP-Se NPs at 4 °C and RT. (**D**) Changes of Zeta potential of SCP-Se NPs at 4 °C and RT.

To investigate the influence of the temperature on the stability of the SCP-Se NPs colloidal solution, the well-prepared colloidal solution from the same reaction was divided into two parts. One was kept in a RT environment, and another was stored at 4 °C. During the monitoring period (lasting for 14 d), the visual changes, particle size, PDI, and Zeta potential of the SCP-Se NPs colloidal solution were recorded. As shown in Figure 7A, the solution remained uniformly dispersed for 14 d when it was kept at 4 °C, while visible precipitation occurred on the 14th day of storage at RT (Figure 7B). Meanwhile, the particle size of SCP-Se NPs at 4 °C was relatively stable, with a particle size of 121 nm on day 0 and 112 nm on day 14, and the PDI remained at a rather low value (Figure 7C). By contrast, the particle size and the PDI of SCP-Se NPs at RT gradually increased to 516 nm and 0.420 on the 14th day, respectively (Figure 7C). Zeta potential results are shown in Figure 7D, and there was no obvious difference between these two different storage conditions.

### 3.6. Effects of SCP-Se NPs on LPS-Induced Enteritis in Mice

**Figure 8:**
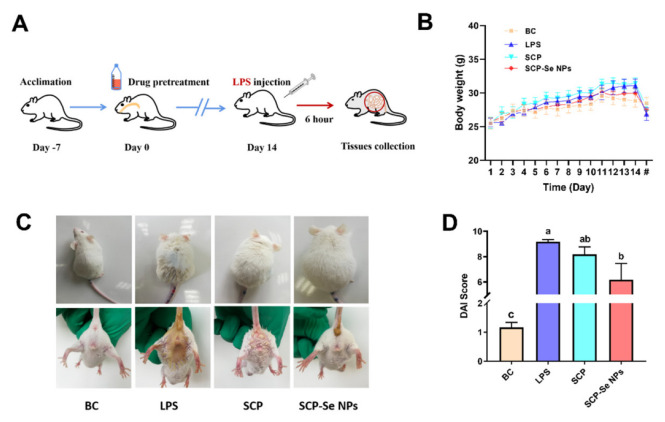
Effects of SCP-Se NPs on LPS-induced enteritis in mice. (**A**) Flow chart of animal experiment. (**B**) Body weight changes of each group during the experiment. “#” indicates the time point of 6 h post-LPS injection. (**C**) Hair and diarrhea in mice after different treatments. (**D**) DAI scores in each group. Different lowercase letters (a–c) indicate significant differences (*p* < 0.05).

The animal experiment flow chart is shown in Figure 8A. During the experiment, mice in the SCP and SCP-Se NPs groups were treated with the corresponding drug solutions for 14 d. As shown in Figure 8B, the body weights of SCP and SCP-Se NPs groups were slightly higher than that of the BC group, but each group was relatively the same amount. After LPS injection, mice in the BC group reacted flexibly and frequently moved with clean and smooth coats and formed stools (Figure 8C). In contrast, mice in the LPS group were depressed, curled together, had bristled hair, diarrhea, perianal contamination, and were positive for fecal occult blood (Figure 8C). Furthermore, the enteritis symptoms of mice treated with SCP and SCP-Se NPs were effectively alleviated (Figure 8C). DAI scoring results are shown in Figure 8D. Among the four groups, the DAI score of the LPS group was the highest, significantly higher than that of the BC group (*p* < 0.05). Meanwhile, compared with the LPS group, the DAI scores of the SCP group and SCP-Se NPs group decreased significantly, and the difference between it and the SCP-Se NPs group was significant (*p* < 0.05). For the SCP-Se NPs group, the enteritis symptoms were reduced, and the DAI score was much lower than that of the SCP group, though there was no significant difference between them (*p >* 0.05).

### 3.7. Effects of SCP-Se NPs on LPS-Induced Intestinal Injuries in Mice

**Figure 9:**
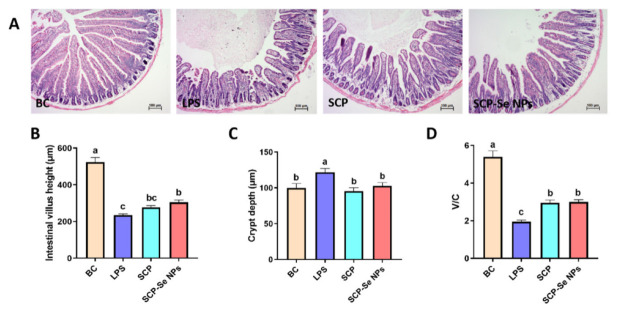
Effect of SCP-Se NPs on LPS-induced intestinal injuries in mice. (**A**) Effect of SCP-Se NPs on the histopathological changes in the jejunum (×100 magnification). (**B**) Jejunum villus height in each group. (**C**) Jejunum crypt depth in each group. (**D**) The ratio of jejunum villus height to crypt depth in each group. Different lowercase letters (a–c) indicate significant differences (*p* < 0.05).

In order to investigate the effects of SCP-Se NPs on LPS-induced intestinal injuries in mice, HE staining was performed on jejunum tissue sections. As shown in Figure 9A, the jejunum villus was complete in structure, orderly arranged, and had no obvious inflammatory cell infiltration in the BC group. Conversely, in the LPS group, a great amount of inflammatory cell infiltration appeared, the jejunum villus structure was destroyed with epithelial cell exfoliation, the villus was broken and atrophied, villus height significantly decreased (*p* < 0.05) (Figure 9B), crypt depth significantly increased (*p* < 0.05) (Figure 9C), and the ratio of villus height to crypt depth significantly decreased (*p* < 0.05) (Figure 9D). Compared with the LPS group, in the SCP and SCP-Se NPs groups, the inflammatory cell infiltration was greatly alleviated, the villus structure was repaired with higher villus height and shallower crypt depth, and the ratio of villus height to crypt depth was significantly elevated (*p* < 0.05) (Figure 9B–D).

### 3.8. Influence of SCP-Se NPs on Jejunum ZO-1 Expression

**Figure 10:**
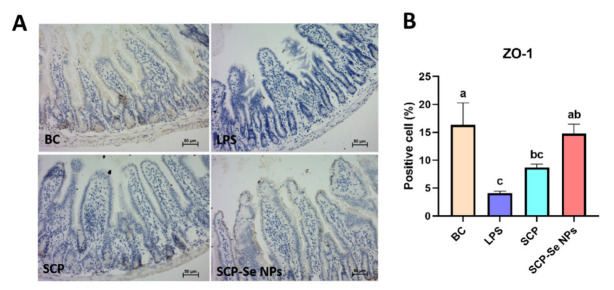
Influence of SCP-Se NPs on ZO-1 expression in LPS-induced intestinal injury. (**A**) IHC image results of jejunum ZO-1 expression in each group (×200 magnification). (**B**) Quantitative analysis of jejunum ZO-1 expression in each group. Different lowercase letters (a–c) indicate significant differences (*p* < 0.05).

The IHC results of jejunum ZO-1 expression in each group are illustrated in Figure 10A. The yellow-brown points indicate the position of the ZO-1 protein. Compared with the BC group, the jejunum ZO-1 expression area of the LPS group was much smaller, and the color of the yellow-brown points was much lighter. Meanwhile, the quantitative analysis results shown in Figure 10B also revealed the significantly decreased expression level of ZO-1 in the LPS group (*p* < 0.05). For the SCP and SCP-Se NPs groups, the ZO-1 expression area was obviously expanded, and the yellow-brown color got darker compared with the LPS group. Importantly, the expression level of ZO-1 in the SCP-Se NPs group was significantly increased (*p* < 0.05), which was close to that of the BC group (Figure 10B).

### 3.9. Effects of SCP-Se NPs on the Expression of Inflammatory Cytokines

**Figure 11:**
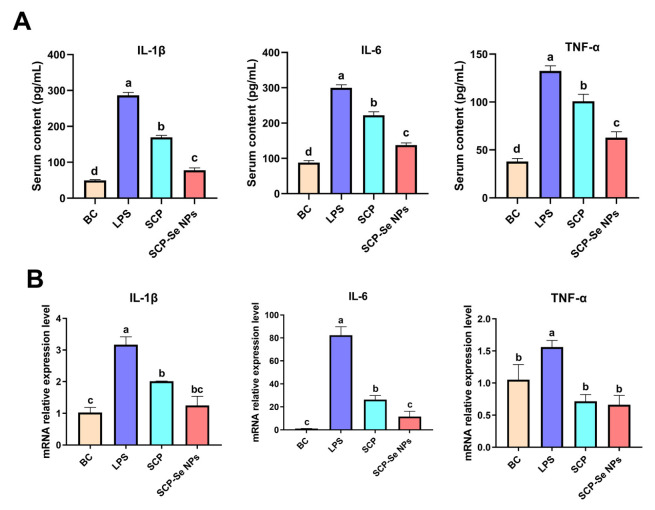
Effects of SCP-Se NPs on inflammatory cytokine (IL-1β, IL-6, and TNF-α) expression levels in LPS-treated mice. (A) Serum inflammatory cytokine (IL-1β, IL-6, and TNF-α) content. (B) Jejunum inflammation cytokine (IL-1β, IL-6, and TNF-α) relative expression level. Different lowercase letters (a–c) indicate significant differences (*p* < 0.05).

To investigate the effects of SCP-Se NPs on alleviating LPS-induced intestinal inflammation, the levels of IL-1β, IL-6, and TNF-α were determined. As shown in Figure 11, the serum concentration and the relative mRNA expression levels of IL-1β, IL-6, and TNF-α of the LPS group were all significantly elevated compared with those of the BC group (*p* < 0.05). Administration of SCP and SCP-Se NPs significantly downregulated the expression of IL-1β, IL-6, and TNF-α (*p* < 0.05). For serum content, all these inflammatory cytokine contents of the SCP-Se NPs group were significantly lower than those of the LPS and SCP groups (*p* < 0.05). For mRNA expression, the IL-6 mRNA expression level of the SCP-Se NPs group was significantly lower than that of the SCP group (*p* < 0.05) and all three of these inflammatory cytokines’ mRNA expression levels of the SCP-Se NPs group were back to normal, showing no significant difference with those of the BC group (*p >* 0.05).

## 4. Discussion

Enteritis is a common disease in animal husbandry, which seriously affects the healthy development of the animal breeding industry. SCP has demonstrated good therapeutic potential on a variety of enteritis diseases [9,10]. However, its macromolecular structural property causing low bioavailability limits its clinical application. Recent studies found that polysaccharides extracted from herbal medicine could be modified with Se NPs to improve their bioactivity and bioavailability [35]. Therefore, SCP-Se NPs were synthesized for the first time by the sodium selenite reduction method, and their anti-enteritis effects were also investigated (Figure 1).

In this experiment, purified SCP was firstly obtained by deproteinization and DEAE-52 cellulose column purification (Figure 2) and then applied to SCP-Se NP synthesis. To investigate the optimal SCP concentration for SCP-Se NP formation, the particle diameter, PDI, and Zeta potential were examined by using dual-wavelength colorimetric and DLS methods. The results showed that bare Se NPs quickly aggregated into black precipitation in the reaction system, the particle diameter and PDI of SCP-Se NPs were the lowest, and the Zeta potential was the highest at the SCP concentration of 0.100 mg/mL (Figure 4). These results demonstrate that SCP acts as a stabilizer, and 0.100 mg/mL SCP is the optimal concentration for SCP-Se NP synthesis. Meanwhile, TEM, XRD, and EDX were applied to further characterize the SCP-Se NPs (Figure 5). We found that the SCP-Se NPs were amorphous, sphere-like particles with a particle diameter of almost 120 nm, consistent with the result detected by DLS (Figure 4C), and the selenium content of SCP-Se was 18.5%, similar to the previous research [36]. These results further confirm the successful synthesis of the SCP-Se NPs. FTIR spectroscopy analysis is an important means to identify the interaction between polysaccharide and Se NPs. Some studies have found that the O-H peak and C-O-H band of some polysaccharide-bound Se NPs did not change significantly, indicating that only a weak interaction between Se and hydroxyl groups occurred without breaking the chemical bond of the hydroxyl group on the Se surface [37]. The red or blue shift could be observed in the O-H peak and C-O-H band of some polysaccharide-bound Se NPs, implying the formation of the new bonds [38,39]. In this study, the hydroxyl peak at 3393 cm^−1^ in the SCP spectrum was red-shifted to 3375 cm^−1^ in the SCP-Se NP spectrum, indicating a reduction of free hydroxyl groups after Se linkage attributed to the formation of O-H···Se bonds. The peak at 1093 cm^−1^ shifted to 1081 cm^−1^, indicating the formation of C-O···Se bonds (Figure 6). This is similar to the combination mode of 1,6-a-D-glucan selenium nanoparticles (CPA-Se NPs) [40]. As shown in Figure 4F, SCP-Se NPs were negatively charged and displayed stability in water. Furthermore, the stability analysis showed that SCP-Se NPs easily aggregated at RT (Figure 7), indicating that the proper storage environment of SCP-Se NPs should be at 4 °C.

Gram-negative bacterial infection is one of the main pathogenic factors of enteritis in livestock and poultry production. LPS is the major cell wall component of the Gram-negative bacteria, commonly used to establish an animal enteritis model of Gram-negative bacterial infection [41,42]. In this study, LPS induced typical symptoms of enteritis, such as diarrhea, weight loss, fecal occult blood, and intestinal tissue damage, indicating the animal intestinal injury model was successfully established (Figure 8 and Figure 9). Interestingly, the symptoms of enteritis and the intestinal pathological injury were obviously attenuated when treated with SCP and SCP-Se NPs, and the effects of SCP-Se NPs were better than SCP (Figure 8 and Figure 9). These results suggest that SCP-Se NPs have better anti-intestinal injury activity than SCP in vivo.

The intestinal barrier plays a key role in preventing pathogen invasion and maintaining intestinal environmental homeostasis. Tight junction proteins (TJP) are the main proteins to maintain the function of the gastrointestinal barrier [43,44,45]. Zonula occludens-1 (ZO-1) is an important member of the TJP family that acts as a scaffold and is the main component of TJPs [46]. Loss of ZO-1 results in a significant increase in the permeability of the colonic mucosa, which allows endotoxin to enter the intestine and leads to marked intestinal inflammation in a mouse model of colitis [47]. In this study, we found that both SCP-Se NPs and SCP treatment effectively alleviated LPS-induced impairment of ZO-1, and the effect of SCP-Se NPs was more pronounced (Figure 10). These results indicate that SCP-Se NPs could more effectively protect the intestinal barrier function than SCP.

When pathogens invade the intestinal cavity of the body, they immediately active the innate immune response by regulating the corresponding signaling pathways, such as JAK-STAT, MAPKs, PI3K-AKT, and NF-κB, to produce a large number of cytokines, such as IL-1β, IL-6, and TNF-α [48]. IL-1, mainly secreted by monocytes, macrophages, and epithelial cells, has strong pro-inflammatory activity and can induce a variety of pro-inflammatory mediators, such as cytokines and chemokines [49]. IL-6 is mainly produced by macrophages and T helper cells and can exert pro-inflammatory functions by activating a variety of target cells, including antigen-presenting cells (APCs) and T cells [50]. TNF-α is one of the earliest and most important mediators of the inflammatory response. During pathogen invasion, intestinal macrophages recruited from blood monocytes rapidly transform into a pro-inflammatory phenotype and produce large amounts of TNF-α once activated [51]. Although the secretion of these cytokines is important for pathogen elimination, the excessive immune response is a key trigger for the impairment of intestinal barrier function. For example, TNF-α can significantly destroy the expression and distribution of TJPs, and IL-1β and IL-6 can not only regulate the reorganization of cytoskeleton proteins, but also can directly lead to the rearrangement of TJPs and weaken the barrier function [52,53,54]. Furthermore, studies have also found that the reduction of IL-1β content in intestinal tissue can improve colitis [55]. Anti-TNF-α targeting therapies can improve the clinical score and mucosal healing in IBD patients [56]. The present study found that treatment of SCP-Se NPs more effectively reduced LPS-induced elevation of IL-1β, IL-6, and TNF-α relative to SCP (Figure 11), closely correlated with its anti-enteritis effects. These results may suggest that SCP-Se NPs can more effectively alleviate the excessive inflammatory response against LPS-induced intestinal injury than SCP, indicating that SCP-Se NPs may serve as good candidates in preventing and treating enteritis in the livestock and poultry industry.

## 5. Conclusions

In conclusion, SCP-Se NPs were synthesized and characterized for the first time and showed a better effect against LPS-induced intestinal injury than SCP, suggesting that SCP-Se NPs may serve as a good candidate for preventing and treating enteritis in the livestock and poultry industry.

## Figures and Tables

**Table 1:** Primer sequence information.

Gene	Primer Sequence (5′-3′)	Product Size (bp)
IL-1β	Forward CACTACAGGCTCCGAGATGAACAAC Reverse TGTCGTTGCTTGGTTCTCCTTGTAC	145 145
IL-6	Forward CTTCTTGGGACTGATGCTGGTGAC Reverse TCTGTTGGGAGTGGTATCCTCTGTG	91 91
TNF-α	Forward CGCTCTTCTGTCTACTGAACTTCGG Reverse GTGGTTTGTGAGTGTGAGGGTCTG	113 113
β-actin	Forward TTCCTTCCTGGGTATGGAAT Reverse GAGGAGCAATGATCTTGATC	206 206

## Data Availability

The data presented in this study are available on request from the corresponding author.

## References

[1] Vancamelbeke M., Vermeire S. (2017). The intestinal barrier: A fundamental role in health and disease. Expert Rev. Gastroenterol. Hepatol..

[2] Camilleri M., Madsen K., Spiller R., Greenwood-Van M.B., Van Meerveld B.G., Verne G.N. (2012). Intestinal barrier function in health and gastrointestinal disease. Neurogastroenterol. Motil..

[3] Tulkens J., Vergauwen G., Van Deun J., Geeurickx E., Dhondt B., Lippens L., De Scheerder M.A., Miinalainen I., Rappu P., De Geest B.G. (2020). Increased levels of systemic LPS-positive bacterial extracellular vesicles in patients with intestinal barrier dysfunction. Gut.

[4] Caradonna L., Amati L., Magrone T., Pellegrino N.M., Jirillo E., Caccavo D. (2000). Enteric bacteria, lipopolysaccharides and related cytokines in inflammatory bowel disease: Biological and clinical significance. J. Endotoxin Res..

[5] Rahimi R., Mozaffari S., Abdollahi M. (2009). On the use of herbal medicines in management of inflammatory bowel diseases: A systematic review of animal and human studies. Dig. Dis. Sci..

[6] Seyed R.H., Homa D. (2011). Herbal plants and their derivatives as growth and health promoters in animal nutrition. Vet. Res. Commun..

[7] Fu J., Li J.X., Sun Y.Z., Liu S., Song F.R., Liu Z.Y. (2023). In-depth investigation of the mechanisms of *Schisandra chinensis* polysaccharide mitigating Alzheimer’s disease rat via gut microbiota and feces metabolomics. Int. J. Biol. Macromol..

[8] Xu M.J., Yan T.X., Gong G.W., Wu B., He B.S., Du Y.Y., Xiao F., Jia Y. (2020). Purification, structural characterization, and cognitive improvement activity of a polysaccharides from *Schisandra chinensis*. Int. J. Biol. Macromol..

[9] Qi Y.L., Chen L.X., Gao K., Shao Z.J., Huo X.H., Hua M., Liu S.X., Sun Y.S., Li S.S. (2019). Effects of *Schisandra chinensis* polysaccharides on rats with antibiotic-associated diarrhea. Int. J. Biol. Macromol..

[10] Su L.L., Mao C.Q., Wang X.C., Li L., Tong H.J., Mao J., Ji D., Lu T.L., Hao M., Huang Z.Y. (2020). The Anti-colitis Effect of *Schisandra chinensis* Polysaccharide Is Associated With the Regulation of the Composition and Metabolism of Gut Microbiota. Front. Cell. Infect. Microbiol..

[11] Tatiana M.G., Sergey V.K., Ivan F.G., Andrey V.K., Anna V.G., Marina I.S., Dmiytiy V.N., Alireza S., Alexander A.M. (2022). Biomedical evaluation of antioxidant properties of lamb meat enriched with iodine and selenium. Open Life Sci..

[12] El-Ghazaly M.A., Fadel N., Rashed E., El-Batal A., Kenawy S.A. (2017). Anti-inflammatory effect of selenium nanoparticles on the inflammation induced in irradiated rats. Can. J. Physiol. Pharmacol..

[13] Zoidis E., Seremelis I., Kontopoulos N., Danezis G.P. (2018). Selenium-Dependent Antioxidant Enzymes: Actions and Properties of Selenoproteins. Antioxidants.

[14] Bahram S., Raveendra R.K., Alexander Y., Adrianna L., Khaled T., Tamiru N.A., Neda B., Wanderly M.Q., Trevor K.S., Shayan S. (2019). Dietary selenium supplementation enhances antiviral immunity in chickens challenged with low pathogenic avian influenza virus subtype H9N2. Vet. Immunol. Immunopathol..

[15] Ala M., Kheyri Z. (2021). The rationale for selenium supplementation in inflammatory bowel disease: A mechanism-based point of view. Nutrition.

[16] Ferreira R.L.U., Sena E.K.C.M., Azevedo E.P., Pinheiro F.I., Cobucci R.N., Pedrosa L.F.C. (2021). Selenium in Human Health and Gut Microflora: Bioavailability of Selenocompounds and Relationship With Diseases. Front. Nutr..

[17] Ahmadi M., Poorghasemi M., Seidavi A., Hatzigiannakis E., Milis C. (2020). An optimum level of nano-selenium supplementation of a broiler diet according to the performance, economical parameters, plasma constituents and immunity. J. Elem..

[18] Ghaderzadeh S., Aghjegheshlagh F.M., Nikbin S., Navidshad B. (2020). Correlation effects of nano selenium and conjugated linoleic acid on the performance, lipid metabolism and immune system of male Moghani lambs. Iran. J. Appl. Anim. Sci..

[19] Verma A.K., Kumar A., Rahal A., Kumar V., Roy D. (2012). Inorganic versus organic selenium supplementation: A review. Pak. J. Biol. Sci..

[20] Arin B., Abhishek B., Sudin B. (2019). Selenium nanoparticles are less toxic than inorganic and organic selenium to mice in vivo. Nucleus.

[21] Alidad B., Ali A.S., Seyed N.M., Mohamad C., Nasser K. (2015). The Effects of Organic, Inorganic, and Nano-Selenium on Blood Attributes in Broiler Chickens Exposed to Oxidative Stress. Acta Sci. Vet..

[22] Hu S.Q., Hu W.C., Li Y.R., Li S.J., Tian H.F., Lu A., Wang J.G. (2020). Construction and structure activity mechanism of polysaccharide nanoselenium carrier. Carbohydr. Polym..

[23] Ren L.R., Wu Z.C., Ma Y., Jian W.J., Xiong H.J., Zhou L.N. (2021). Preparation and growth-promoting effect of selenium nanoparticles capped by polysaccharide-protein complexes on tilapia. J. Sci. Food Agric..

[24] Li J., Shen B.X., Nie S.L., Duan Z.H., Chen K.S. (2019). A combination of selenium and polysaccharides: Promising therapeutic potential. Carbohydr. Polym..

[25] Zhu C.H., Zhang S.M., Song C.W., Zhang Y.B., Ling Q.J., Hoffmann P.R., Li J., Chen T.F., Zheng W.J., Huang Z. (2017). Selenium nanoparticles decorated with *Ulva lactuca* polysaccharide potentially attenuate colitis by inhibiting NF-kB mediated hyper inflammation. J. Nanobiotechnol..

[26] Chen Y., Tang J.B., Wang X.K., Sun F.X., Liang S.J. (2012). An immunostimulatory polysaccharide (SCP-IIa) from the fruit of *Schisandra chinensis* (Turcz.) Baill. Int. J. Biol. Macromol..

[27] Zhu B.Q., Qian C.D., Zhou F.M., Guo J.J., Chen N.P., Gao C.X., Jin B., Ding Z.S. (2020). Antipyretic and antitumor effects of a purified polysaccharide from aerial parts of *Tetrastigma hemsleyanum*. J. Ethnopharmacol..

[28] Wang C.C., Feng L., Su J.Y., Cui L., Liu D., Yan J., Ding C.L., Tan X.B., Jia X.B. (2017). Polysaccharides from *Epimedium koreanum* Nakai with immunomodulatory activity and inhibitory effect on tumor growth in LLC-bearing mice. J. Ethnopharmacol..

[29] Xiao Y.D., Huang Q.L., Zheng Z.M., Guan H., Liu S.Y. (2017). Construction of a *Cordyceps sinensis* exopolysaccharide-conjugated selenium nanoparticles and enhancement of their antioxidant activities. Int. J. Biol. Macromol..

[30] Zhang X., Yan H.H., Ma L., Zhang H.R., Ren D.F. (2020). Preparation and characterization of selenium nanoparticles decorated by *Spirulina platensis* polysaccharide. J. Food Biochem..

[31] Song J.Y., Zhou J.J., Li X., Li P.L., Tian G.Z., Zhang C., Zhou D.Z. (2022). Nano-selenium stablilized by Konjac Glucommannan and its biological activity in vitro. LWT-Food Sci. Technol..

[32] Keyhani A., Mahmoudvand H., Shakibaie M. (2018). Histopathological and toxicological study of selenium nanoparticles in BALB/C Mice. Entomol. Appl. Sci. Lett..

[33] Liang Y.C., Liu H.J., Chen S.H., Chen C.C., Chou L.S., Tsai L.H. (2005). Effect of lipopolysaccharide on diarrhea and gastrointestinal transit in mice: Roles of nitric oxide and prostaglandin E_2_. World J. Gastroenterol..

[34] Liu Q., Peng Z.M., Zhou L., Peng R.Q., Li X.H., Zuo W., Gou J.H., Zhou F.X., Yu S.J., Huang M. (2022). Short-Chain Fatty Acid Decreases the Expression of CEBPB to Inhibit miR-145-Mediated DUSP6 and Thus Further Suppresses Intestinal Inflammation. Inflammation.

[35] Shi X.D., Tian Y.Q., Wu J.L., Wang S.Y. (2021). Synthesis, characterization, and biological activity of selenium nanoparticles conjugated with polysaccharides. Crit. Rev. Food Sci. Nutr..

[36] Cui D.X., Ma J., Liang T.T., Sun L.Q., Meng L.Q., Liang T.G., Li Q.S. (2019). Selenium nanoparticles fabricated in laminarin polysaccharides solutions exert their cytotoxicities in HepG2 cells by inhibiting autophagy and promoting apoptosis. Int. J. Biol. Macromol..

[37] Cai W.F., Hu T., Amr M.B., Zheng Z.M., Xiao Y.D., Huang Q.L. (2018). Effect of ultrasound on size, morphology, stability and antioxidant activity of selenium nanoparticles dispersed by a hyperbranched polysaccharide from *Lignosus rhinocerotis*. Ultrason. Sonochem..

[38] Wang L., Li C., Huang Q., Fu X. (2019). Biofunctionalization of selenium nanoparticles with a polysaccharide from *Rosa roxburghii* fruit and their protective effect against H_2_O_2_-induced apoptosis in INS-1 cells. Food Funct..

[39] Zhang S.J., Song Z.T., Shi L.J., Zhou L.N., Zhang J., Cui J.L., Li Y.H., Jin D.Q., Ohizumi Y., Xu J. (2021). A dandelion polysaccharide and its selenium nanoparticles: Structure features and evaluation of anti-tumor activity in zebrafish models. Carbohydr. Polym..

[40] Li H.Y., Liu D.D., Li S.H., Xue C.H. (2019). Synthesis and cytotoxicity of selenium nanoparticles stabilized by α-D-glucan from *Castanea mollissima* Blume. Int. J. Biol. Macromol..

[41] Zhang L.L., Wei X.B., Zhang R.J., Si D.Y., James N.P., Baseer A., Zhang M.Y. (2019). A Novel Peptide Ameliorates LPS-Induced Intestinal Inflammation and Mucosal Barrier Damage via Its Antioxidant and Antiendotoxin Effects. Int. J. Mol. Sci..

[42] Wang X.Y., Wang W.J., Wang L.M., Yu C., Zhang G.L., Zhu H.L., Wang C.W., Zhao S.J., Hu C.A.A., Liu Y.L. (2019). Lentinan modulates intestinal microbiota and enhances barrier integrity in a piglet model challenged with lipopolysaccharide. Food Funct..

[43] Liu J.L., Gao R.R., Gu X.J., Yu B., Wu Y., Li Q.S., Xiang P., Xu H. (2022). A New Insight into Toxicity of Colchicine Analogues by Molecular Docking Analysis Based on Intestinal Tight Junction Protein ZO-1. Molecules.

[44] Oshima T., Miwa H. (2016). Gastrointestinal mucosal barrier function and diseases. J. Gastroenterol..

[45] Zachary M.S., Anthony T.B. (2020). The Integral Role of Tight Junction Proteins in the Repair of Injured Intestinal Epithelium. Int. J. Mol. Sci..

[46] Ikenouchi J., Umeda K., Tsukita S., Furuse M., Tsukita S. (2007). Requirement of ZO-1 for the formation of belt-like adherens junctions during epithelial cell polarization. J. Cell Biol..

[47] Poritz L.S., Garver K.I., Green C., Fitzpatrick L., Ruggiero F., Koltun W.A. (2007). Loss of the Tight Junction Protein ZO-1 in Dextran Sulfate Sodium Induced Colitis. J. Surg. Res..

[48] Wei J., Feng J.X. (2010). Signaling pathways associated with inflammatory bowel disease. Recent Pat. Inflamm. Allergy Drug Discov..

[49] Gabay C., Lamacchia C., Palmer G. (2010). IL-1 pathways in inflammation and human diseases. Nat. Rev. Rheumatol..

[50] Neurath F.M. (2014). Cytokines in inflammatory bowel disease. Nat. Rev. Immunol..

[51] Konstantinos H.K., Papadakis K.A. (2017). Inflammatory Bowel Disease: Updates on Molecular Targets for Biologics. Gut Liver.

[52] Kim K.Y., Oh T.W., Do H.J., Yang J.H., Yang I.J., Jeon Y.H., Go Y.H., Ahn S.C., Ma J.Y., Park K.I. (2018). Acer palmatum thumb. ethanol extract alleviates interleukin-6-induced barrier dysfunction and dextran sodium sulfate-induced colitis by improving intestinal barrier function and reducing inflammation. J. Immunol. Res..

[53] Zhou H.Y., Zhu H., Yao X.M., Qian J.P., Yang J., Pan X.D., Chen X.D. (2017). Metformin regulates tight junction of intestinal epithelial cells via MLCK-MLC signaling pathway. Eur. Rev. Med. Pharmacol. Sci..

[54] Wu Y.P., Zhu C., Chen Z., Chen Z.J., Zhang W.N., Ma X.Y., Wang L., Yang X.F., Jiang Z.Y. (2016). Protective effects of *Lactobacillus plantarum* on epithelial barrier disruption caused by enterotoxigenic *Escherichia coli* in intestinal porcine epithelial cells. Vet. Immunol. Immunopathol..

[55] Loris R.L., Chowdhry S., Pizarro T.T. (2013). Opposing functions of classic and novel IL-1 family members in gut health and disease. Front. Immunol..

[56] Matthias F., Mathilde P., Fiona P. (2019). Cytokine Networks in the Pathophysiology of Inflammatory Bowel Disease. Immunity.

